# Curcumin Mitigates the Intracellular Lipid Deposit Induced by Antipsychotics *In Vitro*

**DOI:** 10.1371/journal.pone.0141829

**Published:** 2015-10-30

**Authors:** Alberto Canfrán-Duque, Oscar Pastor, Manuel Reina, Milagros Lerma, Alfonso J. Cruz-Jentoft, Miguel A. Lasunción, Rebeca Busto

**Affiliations:** 1 Servicio de Bioquímica-Investigación, Hospital Universitario Ramón y Cajal, IRyCIS, Madrid, Spain; 2 Servicio de Bioquímica-Clínica, Hospital Universitario Ramón y Cajal, IRyCIS, Madrid, Spain; 3 Celltec-UB, Department of Cell Biology, University of Barcelona, Barcelona, Spain; 4 Unidad de Geriatría, Hospital Universitario Ramón y Cajal, IRyCIS, Madrid, Spain; 5 CIBER Fisiopatología de la Obesidad y Nutrición (CIBEROBN), Instituto de Salud Carlos III (ISCIII), Madrid, Spain; Albert Einstein College of Medicine, UNITED STATES

## Abstract

**Scope:**

First- and second-generation antipsychotics (FGAs and SGAs, respectively), both inhibit cholesterol biosynthesis and impair the intracellular cholesterol trafficking, leading to lipid accumulation in the late endosome/lysosome compartment. In this study we examined if curcumin, a plant polyphenol that stimulates exosome release, can alleviate antipsychotic-induced intracellular lipid accumulation.

**Methods:**

HepG2 hepatocarcinoma cells were treated with antipsychotics or placebo and DiI-labelled LDL for 18 h and then exposed to curcumin for the last 2 h. Cells and media were collected separately and used for biochemical analyses, electron microscopy and immunocytochemistry. Exosomes were isolated from the incubation medium by ultracentrifugation.

**Results:**

Curcumin treatment reduced the number of heterolysosomes and shifted their subcellular localization to the periphery, as revealed by electron microscopy, and stimulated the release of lysosomal β-hexosaminidase and exosome markers flotillin-2 and CD63 into the media. The presence of DiI in exosomes released by cells preloaded with DiI-LDL demonstrated the endolysosomal origin of the microvesicles. Furthermore, curcumin increased the secretion of cholesterol as well as LDL-derived DiI and [^3^H]-cholesterol, in association with a decrease of intracellular lipids. Thus, the disruption of lipid trafficking induced by FGAs or SGAs can be relieved by curcumin treatment. This polyphenol, however, did not mitigate the reduction of cholesterol esterification induced by antipsychotics.

**Conclusion:**

Curcumin stimulates exosome release to remove cholesterol (and presumably other lipids) accumulated within the endolysosomal compartment, thereby normalizing intracellular lipid homeostasis. This action may help minimize the adverse metabolic effects of antipsychotic treatment, which should now be evaluated in clinical trials.

## Introduction

Antipsychotic drugs are widely used to alleviate a number of psychiatric disorders. There are two classes of antipsychotic drugs, typical or first-generation antipsychotics (FGAs) and atypical or second generation antipsychotics (SGAs). The therapeutic efficacy of both classes is strongly associated with antagonism of D_2_ dopamine receptors [1], SGAs exhibiting lower affinity for D_2_ receptors and greater affinity to serotonergic, histaminergic, muscarinic, and adrenergic receptors than FGAs [1]. Both classes of antipsychotics are associated with serious side effects. The most salient side effects of most antipsychotic are acute and chronic extrapyramidal symptoms or movement disorders. The SGAs show reduced incidence of such neurological side effects but the blockade of other neurotransmitter receptors is thought to underline other adverse effects [2,3]. Recently, a higher risk of stroke in antipsychotic users has been reported, especially in older patients with vascular illness, although the pathophysiology of such association is still unclear [4].

Antipsychotics have been also shown also to disrupt lipid homeostasis [5–9]. Both FGAs and SGAs inhibit cholesterol biosynthesis *in vitro* by altering the activities of various enzymes in the biosynthesis pathway, resulting in the accumulation of different sterol intermediates (the substrates of the inhibited enzymes) depending on the specific drug and dose [5,6,8,10]. This alteration in sterol profile affects lipid raft formation and consequently cell signaling triggered by hormones as demonstrated for insulin and somatostatin in cells treated with haloperidol [5,10].

Antipsychotics also impair intracellular cholesterol trafficking and interfere with low-density lipoprotein (LDL)-derived cholesterol egress from the endosome/lysosome compartment, thereby reducing the arrival of endocytosed LDL cholesterol to both the endoplasmic reticulum (ER) [5,6,8] and the trans-Golgi [11]. Cholesterol reaching the ER can be esterified as a storage form and/or sensed by the sterol-regulatory-element-binding protein (SREBP)-Scap-Insig system [12]. The reduction in ER cholesterol is in accordance with the observed upregulation of SREBP and downstream target genes in response to antipsychotic treatment [8,13]. Given that excess free cholesterol is cytotoxic, lipid levels within the cell are tightly regulated. Complex mechanisms have evolved to regulate cellular lipid abundance and distribution between cell compartments by a feedback pathway that controls the synthesis, esterification, uptake, and efflux of cholesterol [14]. However, exposure to antipsychotics has been shown to alter intracellular lipid homeostasis, as it reduces cholesterol availability in the ER, increases LDL endocytosis, and stimulates fatty acid and triglyceride biosynthesis [8].

Curcumin, the main active polyphenol extracted from the rhizome of *Curcuma longa* (Turmeric), has multiple beneficial effects against inflammation, hyperlipidemia, and atherosclerosis [15]. The mechanisms by which curcumin affects lipid metabolism appears to be diverse [16]. Of noting, curcumin has been shown to potently elevate cytosolic free Ca^2+^ levels by inhibiting sarco/endoplasmic reticulum Ca^2+^-ATPase (SERCA) activity [17], which may affect multiple cellular processes, including enzyme activity and vesicular trafficking [18,19]. In particular, regarding the intracellular lipid traffic, curcumin alleviated the accumulation of cholesterol, sphingomyelin, and glycosphingolipids in the endocytic compartment characteristic of Niemann Pick disease type C (NPC) by restoring endocytic calcium depletion [20]. Moreover, curcumin has been shown to stimulate LDL receptor (LDLR) at the levels of mRNA, protein and activity in different cell types, included hepatocytes, lymphocytes, macrophages and renal cells [21,22] although a suppression of *LDLR* gene expression was observed in stellate cells [23]. Curcumin also stimulates cholesterol efflux mediated by ATP-binding cassette protein A1 (ABCA1) in adipocytes [24] and macrophages [25]. Finally, curcumin has been shown to stimulate exosome secretion in a cellular model of NPC1 deficiency, thus reducing the intracellular cholesterol accumulation [26].

Exosomes are small vesicles secreted by most cell types in culture. They are formed inside the cell by inward budding of the limiting membranes of endocytic compartments, creating vesicle-containing endosomes termed multivesicular bodies (MVBs). These MVBs can fuse with the plasma membrane, thereby releasing their internal vesicles, exosomes, into the extracellular space [27]. The secretion of exosomes is triggered by cytoplasm Ca^2+^ [28,29]. Exosomes are involved in numerous physiological and pathological processes, including cell-cell communication by horizontal transfer of proteins and RNA, antigen presentation, tumor metastasis, propagation of infectious agents, and release of superfluous membranes and cytosol [30]. In addition, exosome release could function to eliminate undesired materials from the cell, such as excess lipids [31]. Both oligodendrocytes treated with U18666A and NPC1-deficient fibroblasts combated cholesterol accumulation in the lysosomal compartment by increasing the secretion of exosomes [32]. However, the potential of curcumin to attenuate antipsychotic-induced disruption in lipid homeostasis by exosomal release has not been examined. Here we show that curcumin promotes exosome release, thus reducing the late-endosome/lysosome trapping of cholesterol induced by antipsychotic drugs.

## Materials and Methods

All chemicals were purchased from Sigma (Sigma-Aldrich Química, S.A., Tres Cantos, Madrid, Spain) unless otherwise stated. The antipsychotics used were clozapine free base, haloperidol free base, risperidone free base, and ziprasidone hydrochloride (Tocris, Bristol, United Kingdom).

### Cell culture

HepG2 hepatocarcinoma cells (ATCC HB-8065) (Rockville, MD, USA) were cultured in high-glucose DMEM (Gibco, Life Technologies Corporation, Madrid, Spain) supplemented with MEM non-essential amino acids, 10% fetal bovine serum (FBS), and antibiotics (Gibco) at 37°C in a 5% CO_2_ atmosphere. Lipoprotein-deficient serum (LPDS) was prepared from FBS by ultracentrifugation at a density of 1.21 kg/L. Cells were cultured in medium with 10% LPDS supplemented with LDL (60 μg/ml of cholesterol), and then incubated for 16 h with no drug (control) or with an antipsychotic (clozapine, haloperidol, risperidone, or ziprasidone at 10 μM). Cells were then washed twice with PBS and incubated in serum-free medium with the same antipsychotic with or without curcumin (30 μM) for an additional 2 h. Drugs were dissolved in DMSO (dimethyl sulfoxide; final concentration in the medium, 0.044%). Control cells were exposed to the solvent. Human LDL, isolated as described [33], was labeled with the fluorescent probe 1,1′-dioctadecyl-3,3,3,3′-tetramethylindocarbocyanineperchlorate (DiI, Life Technologies Corporation) as previously described [34]. Cell viability was determined using XTT assays (cell proliferation kit; Roche, Mannheim, Germany).

### Immunofluorescence microscopy

HepG2 cells were cultured on glass coverslips, treated as indicated, and fixed with 4% paraformaldehyde (PFA)/PBS for 5 min. Fixed cells were permeabilized in 0.1% Triton X-100/PBS for 5 min and blocked with 2% bovine serum albumin in PBS for 45 min. For free cholesterol staining, cells were exposed to filipin (50 mg/L in PBS) for 45 min. Cells were stained with anti-CD63 (1/200, Ref 55619, BD Biosciences, Madrid, Spain) at a 1:200 dilution for 2 h, followed by incubation with Alexa fluor-conjugated IgG (Molecular Probes, Life Technologies Corporation) in PBS at a 1:500 dilution for 45 min. Cells were mounted for microscopy and examined on a Nikon D Eclipse C1 confocal microscope.

### Intracellular DiI-LDL accumulation by flow cytometry

DiI-LDL uptake was assayed as previously described [35]. Cells were incubated with DiI-LDL (30 μg cholesterol/ml) and the intracellular content was analyzed by a FACSscan flow cytometry system (BD Biosciences). Non-specific accumulation was determined in extra tubes containing DiI-LDL and a 50-fold excess of unlabelled LDL. Results are expressed as specific median intensity of fluorescence (M.I.F.) after subtracting autofluorescence from cells incubated in the absence of DiI-LDL.

### Cholesterol efflux assays

To measure cholesterol efflux, LDL was labeled with [^3^H]-cholesterol (Perkin-Elmer, Shelton, CT, USA) following a previously published method [36] with minor modifications. Briefly, 2 ml of LDL (5 mg/ml) in 0.9% NaCl was added to [^3^H]-cholesterol (200 μCi; dried with N_2_) and incubated at 4°C for 5–7 h with occasional mixing. Cells in 10% LPDS-containing medium were incubated with [^3^H]-cholesterol-labeled LDL (60 μg/ml of cholesterol) for 16 h with or without an antipsychotic (clozapine, haloperidol, risperidone, or ziprasidone at 10 μM). The cells were then washed twice with PBS and cultured for 2 additional hours in serum-free medium with the same antipsychotic with or without curcumin. The culture medium was harvested and the cell monolayer washed with PBS and treated with 0.1 M NaOH to extract total intracellular [^3^H]-radioactivity. Radioactivity was counted with a liquid scintillation analyzer (Tri-Carb 2800TR, Perkin Elmer). The cholesterol efflux was calculated as the percent of [^3^H]-cholesterol released into the medium.

### Analysis of cholesterol by gas chromatography/mass spectroscopy (GC/MS)

Sterols were extracted using chloroform:methanol (2:1, vol/vol) and analyzed using an Agilent 6890N GC and an Agilent 5975 MS detector (Agilent Technologies, Santa Clara, CA, United States) with an Agilent DB-5ms, 30 m × 0.25 mm × 0.1 μm column [5,8]. For quantitation, the MS detector was operated in selective ion monitoring (SIM) mode following at least one quantifier and two qualifying ions for each sterol. Peak identification was achieved both by comparison with known external standards and by monitoring characteristic ions.

### Measurement of [^3^H]oleic acid incorporation into cholesteryl esters

The cells in 10% LPDS-containing medium were incubated for 16 h with or without an antipsychotic (clozapine, haloperidol, risperidone, or ziprasidone at 10 μM). Then they were washed twice with PBS and cultured for 2 additional hours in serum-free medium with the same antipsychotic with or without curcumin. An emulsion of 1.3 μCi [9,10-^3^H]oleic acid per well (50 Ci/mmol, Hartmann Analytic GmbH, Braunschweig Germany) containing 0.15% human serum albumin, was added to the medium the last 2 h. Then the lipids extracts were analyzed by high performance liquid chromatography (HPLC) using an Agilent HPLC system consisted of a 1260 LC quaternary pump a 1260 injector, a 1260 oven set at 40°C, and a Agilent 1200 evaporative light scattering detector set to gain 4, drift tube temperature 45°C and 3.0 bar internal nitrogen pressure with a Kinetex-HILIC column (2.6 μm, 100 x 46 mm, Phenomenex) and a guard cartridge (HILIC, 4.6 mm, Phenomenex) as described in detail by [37]. The cholesterol ester fraction was collected, dried and the radioactivity was counted.

### Exosome isolation and analysis

Exosomes were isolated from culture media as previously described [38]. Briefly, after treating the cells (5 × 10^6^/dish), the medium was collected on ice, centrifuged at 800 × g for 10 min to sediment cells and then twice at 12,000 × g for 30 min to sediment debris. Exosomes were sedimented from the remaining supernatant by ultracentrifugation at 100,000 × g for 2 h. The exosome pellet was resuspended in PBS. The purity of the exosome preparation was evaluated by analyzing the content of an ER marker, calnexin, by Western blot, which was barely detected in every exosome preparation [26].

Late endosome exocytosis was quantitated by measuring the activity of β-N-acetylglucosaminidase (β-hexosaminidase), a lysosomal enzyme, in the medium of HepG2 cells using a β-N-acetylglucosaminidase assay kit. Enzyme released into the medium was expressed as a percentage of the total (medium plus that in lysed cells).

For Western blot analysis, cell lysates were loaded at 20–30 μg protein per gel lane and exosome samples at the maximum lane well volume. Samples were solubilized in reducing SDS loading buffer, separated on 8%–10% SDS-PAGE, and transferred to nitrocellulose membranes (Bio Rad Laboratories S.A., Barcelona, Spain). After blocking, the membranes were probed with specific antibodies for flotillin-2 (1/500, Ref 610383 BD Biosciences), CD63 (1/300, H193), and β-actin (1/3000, I-19) as the gel loading control (Santa Cruz Biotechnology), followed by incubation with secondary antibodies conjugated to IRDye 800CW or IRDye 680LT (LI-COR, Lincoln, NE, USA) in the dark. Immunolabeling was detected using the Odyssey Infrared Imaging System (LI-COR).

### Exosome bead fluorescence-activated cell sorting (FACS)

Exosomes obtained from HepG2 cells incubated with DiI-LDL were absorbed onto 4 μm aldehyde-sulfate latex beads (Invitrogen, Life Technologies Corporation) and incubated first with CD63 antibody (1/200, Ref 55619, BD Biosciences) and then with a fluorophore-conjugated secondary antibody. Cells were washed and analyzed by flow cytometry (FACSscan, BD Biosciences) as previously described [39].

### Electron microscopy

Following drug treatment, cells were fixed in 2% glutaraldehyde for 1 h and with 4% PFA for 1 h. Fixed cells were washed with sodium phosphate buffer, post-fixed with 1% osmium tetroxide for 1 h, dehydrated in increasing concentrations of ethanol, and embedded in epoxy resin (Fluka, Sigma-Aldrich). Ultrathin sections were prepared, stained with uranyl acetate and lead citrate, and examined under a JEOL JEM1010 transmission electron microscope (JEOL Ltd., Tokyo, Japan).

Exosome preparations were fixed in 2% PFA and loaded onto Formvar^®^ carbon coated grids (Thermo Fisher Scientific, Rochester, NY, USA). Then, the exosomes were post-fixed in 1% glutaraldehyde, washed, contrasted in 2% uranyl acetate, embedded in a mixture of uranyl acetate (0.8%) and methyl cellulose (2%), and examined in a JEOL JEM1010 electron microscope.

### Statistical Analysis

Data are shown as mean ± SEM. Treatment group means were compared by two-way repeated measures ANOVA followed by post hoc Bonferroni tests for multiple comparisons using GraphPad Prism 6 software. In all cases, a confidence level of 5% was considered statistically significant (*P* < 0.05).

## Results

### Changes in HepG2 cell ultrastructure induced by antipsychotics and mitigation by curcumin

In this study we used the HepG2 cells for the intense lipid metabolism of hepatocytes and the involvement of liver in the metabolism of antipsychotics. Antipsychotic interfere with LDL-derived cholesterol egress from the endosome/lysosome compartment and thereby induced intracellular lipid accumulation [5,8,10]. To determine whether curcumin alleviates the intracellular lipid accumulation induced by antipsychotics, in preliminary experiments we analyzed the effects of different concentrations of curcumin on the intracellular accumulation of LDL induced by 10 μM haloperidol. For this, the cells were incubated in the presence of DiI-LDL for 16 h and, then the medium was replaced by a fresh one without DiI-LDL and the cells were incubated with curcumin (5, 10, 30 and 40 μM) for additional 2 h, always in the absence (control) or the presence of haloperidol (Fig A in S1 File). The results revealed that 30 and 40 μM of curcumin concentration reduced DiI-LDL accumulation in cells treated with haloperidol (Fig A in S1 File). Therefore we decided to select the dose of 30 μM curcumin for the following experiments.

The HepG2 cells were incubated in medium containing LDL and one of the following antipsychotics (10 μM): haloperidol (an FGA), clozapine, risperidone, or ziprasidone (SGAs) during 18 h. Where indicated, cells were treated with curcumin (30 μM) for the last 2 h of incubation. Treatment of HepG2 cells without (control) or with 30 μM curcumin (2 h) had no appreciable effect on cell proliferation as measured by the XTT assay (Control = 100.0 ± 4.5 and curcumin = 100.6±3.0 a.u.f.; mean ± SEM, n = 3).

**Fig 1:**
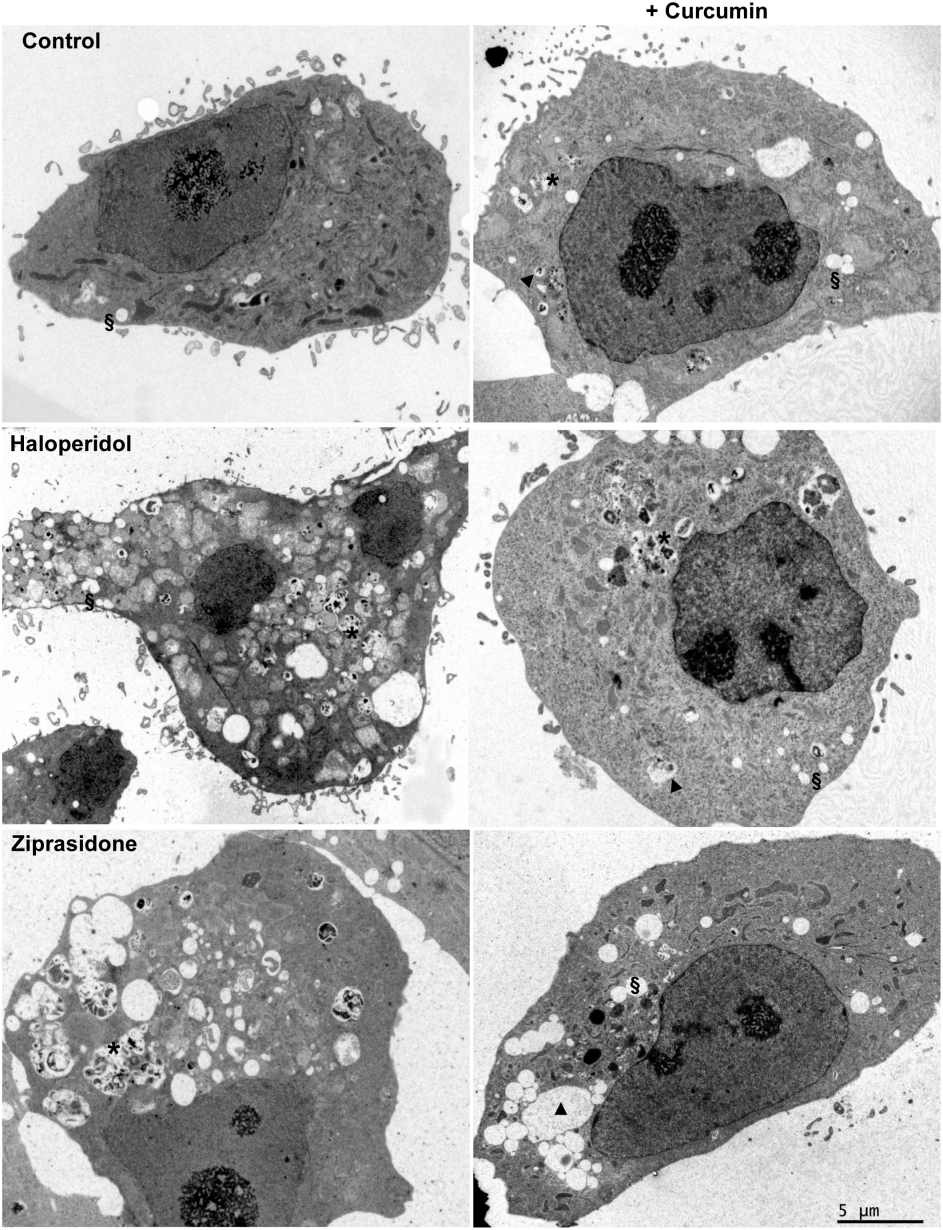
Electron micrographs of HepG2 cells untreated (control) or treated with an antipsychotic (haloperidol or ziprasidone at 10 μM) and LDL (60 μg/ml of cholesterol) for a total of 18 h. Where indicated, during the last 2 h, the cells were treated with 30 μM curcumin. Images are representative from 2 independent experiments. Lysosomes (▲), lipid inclusions (**§**), heterolysosomes or MVB (*).

Ultrastructural analysis of untreated control HepG2 cells revealed relatively few lysosomes and marked morphological heterogeneity of mitochondria (Fig 1). In general, the main effect of antipsychotic treatment was the increase in the number of heterolysosomes or MVB. Lipid inclusions, secondary lysosomes, and abnormal mitochondria also were observed (Fig 1 and Fig B in S1 File). These results are in accordance with our previous fluorescence microscopy study showing altered intracellular LDL trafficking in HepG2 cells treated with FGAs or SGAs [5,8]. Subsequent treatment with curcumin for 2 h in the continued presence of the antipsychotic reduced the number of heterolysosomes and secondary lysosomes (Fig 1 and Fig B in S1 File). Altogether, the results suggested that curcumin promotes the removal of material accumulated within heterolysosomes.

### Mitigation of the antipsychotic-induced lysosomal accumulation of cholesterol by curcumin

**Fig 2:**
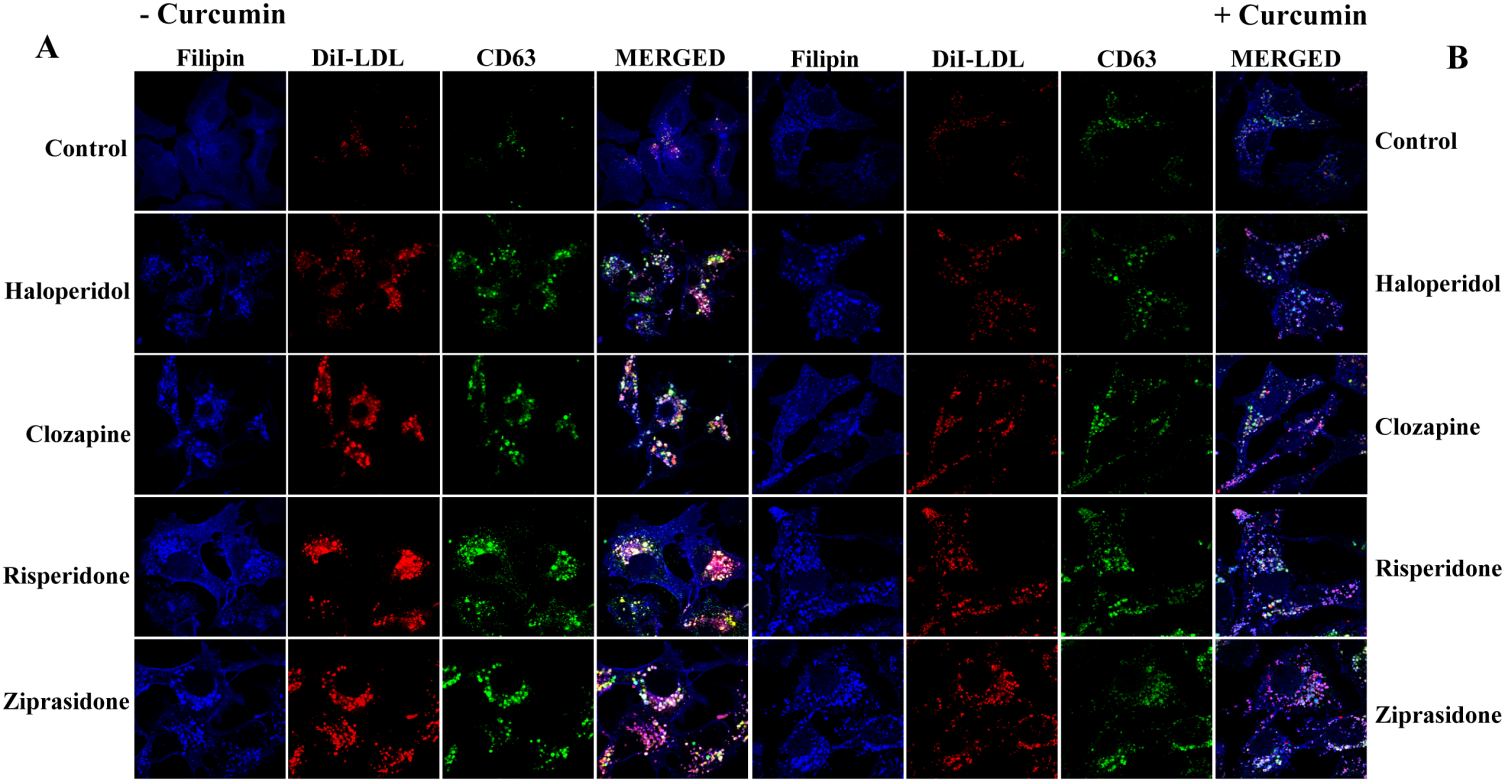
Effects of curcumin on the intracellular distribution of LDL in cells treated with antipsychotics. (A) HepG2 cells were exposed to DiI-LDL (30 μg/ml of cholesterol) in the absence (control) or the presence of antipsychotic (haloperidol, clozapine, risperidone, or ziprasidone, 10 μM) for a total of 18 h. The cells were then washed, fixed, stained with filipin and anti-CD63, and analyzed by confocal microscopy. Representative results from 3 independent experiments are shown. (B) Cells were exposed to DiI-LDL in the absence (control) or the presence of antipsychotic (10 μM) for a total of 18 h and curcumin (30 μM) was added for the last 2 h. The cells were then washed, fixed, stained with filipin and anti-CD63, and analyzed by confocal microscopy. Representative results from 3 independent experiments are shown.

**Fig 3:**
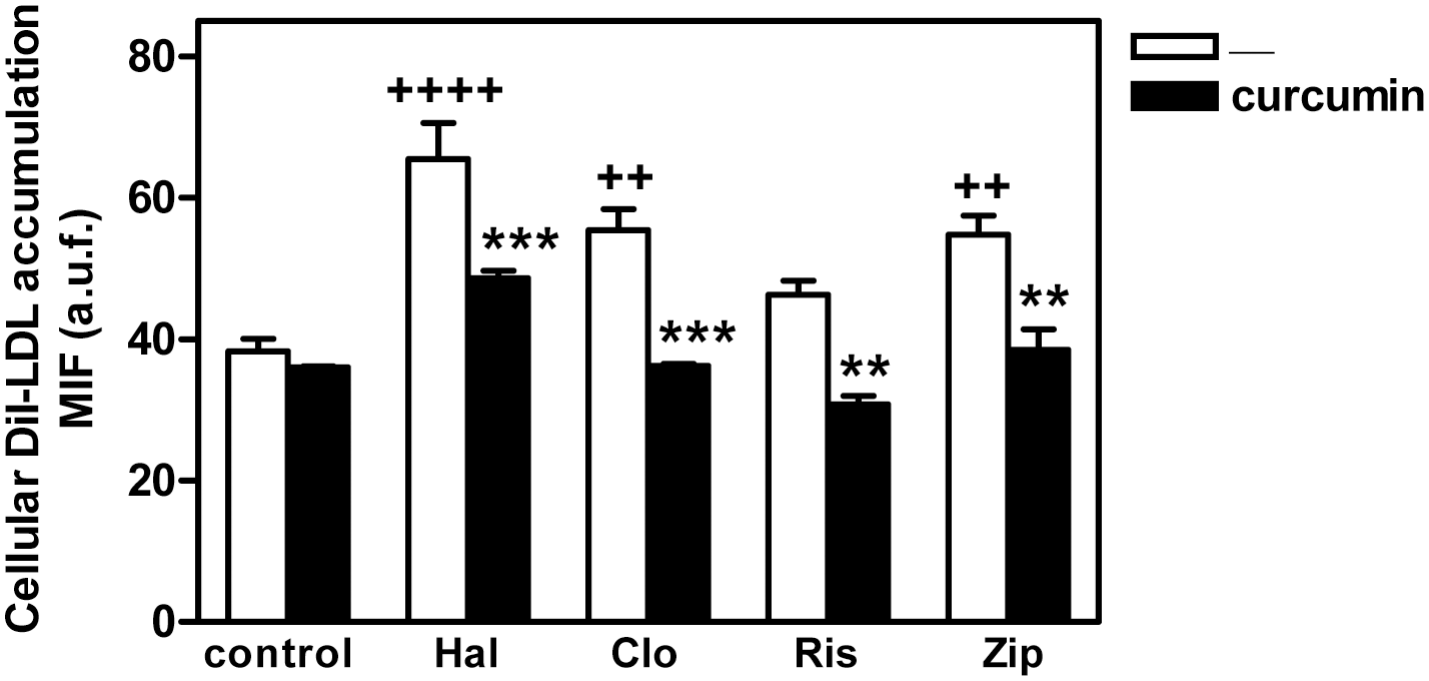
Effect of curcumin on the intracellular DiI-LDL accumulation in cells treated with antipsychotics. HepG2 cells were incubated with DiI-LDL (30 μg/ml of cholesterol) for 16 h in the absence (control) or the presence of haloperidol (Hal), clozapine (Clo), risperidone (Ris), or ziprasidone (Zip) (10 μM). Then, cells were washed and serum-free medium containing the same antipsychotic with or without 30 μM curcumin was added and incubated for additional 2 h. Intracellular DiI-LDL was measured by flow cytometry. Results are mean ± SEM of three independent experiments performed in duplicate. M.I.F., median intensity of fluorescence; a.u.f., arbitrary units of fluorescence. Statistical comparisons shown are curcumin versus no curcumin (* *P*<0.05, ** *P*<0.01 and *** *P*<0.001) and antipsychotic versus untreated control (^+^ *P*<0.05, ^++^ *P*<0.01, ^+++^ *P*<0.001 and ^++++^ *P*<0.0001).

We thus analyzed the effects of curcumin on the intracellular accumulation of LDL induced by the antipsychotics. For this, the cells were incubated in the presence of DiI-LDL for 16 h and, then the medium was replaced by a fresh one without DiI-LDL and the cells were incubated with curcumin 30 μM for additional 2 h, always in the absence (control) or the presence of the antipsychotic. Cells treated with antipsychotics exhibited increased filipin staining in bright perinuclear granules compared to the control, indicative of cholesterol accumulation. These granules were positive for CD63 (Fig 2A) and contained DiI, indicating that the free cholesterol was derived from internalized DiI-LDL and accumulated in the late endosome/lysosome compartment. When cells were challenged with curcumin for the last 2 h, a decrease in the size of granules positive for DiI, filipin, and CD63 was observed in cells treated with antipsychotics. Of note, abundant DiI- and filipin-positive vesicles appeared at the cell periphery (Fig 2B). Studies by flow cytometry revealed that curcumin reduced DiI-LDL accumulation in cells treated with an antipsychotic (Fig 3), confirming that this polyphenol ameliorates the intracellular cholesterol trafficking block.

### Curcumin enhanced cholesterol secretion from antipsychotic-treated cells

**Fig 4:**
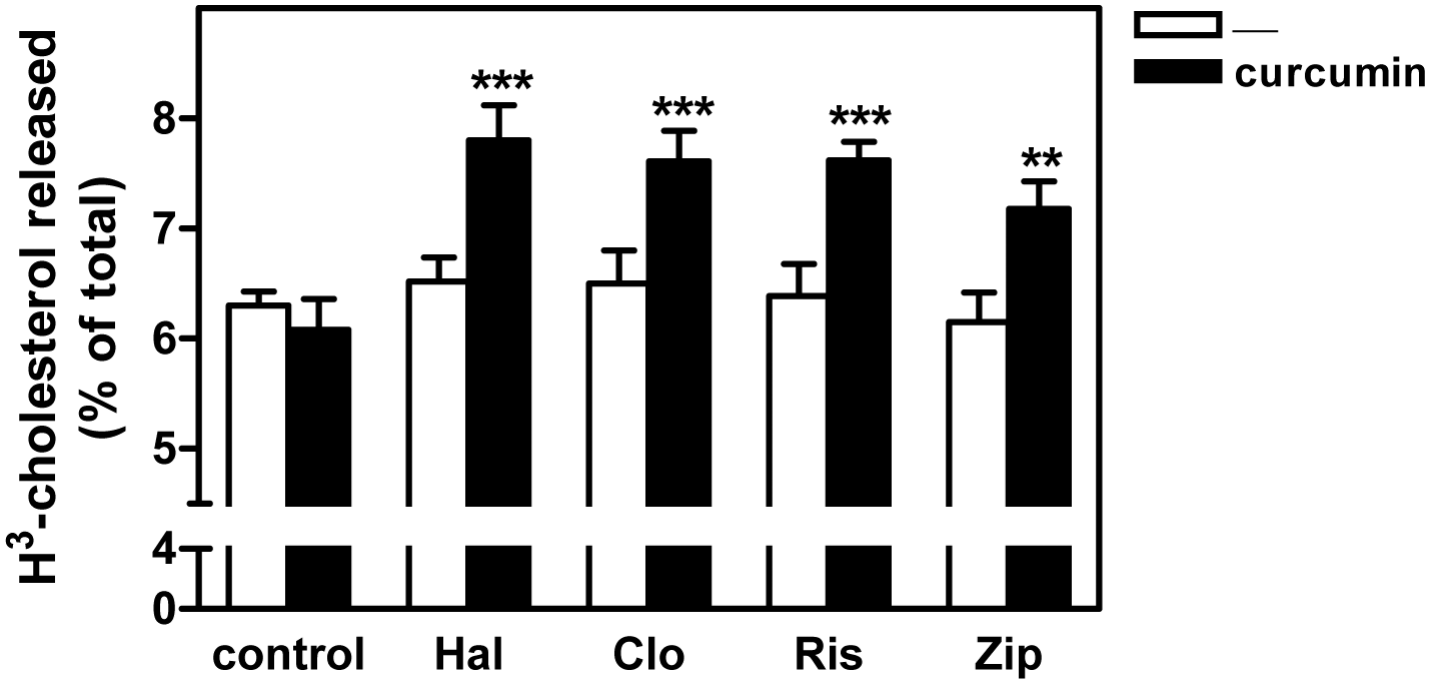
Curcumin increases LDL-derived cholesterol efflux in HepG2 cells treated with antipsychotics. HepG2 cells were exposed to ^3^H-cholesterol-labeled LDL (60 μg/ml of cholesterol) for 16 h in the absence (control) or presence of haloperidol (Hal), clozapine (Clo), risperidone (Ris), or ziprasidone (Zip) (10 μM). Cells were washed twice and serum-free medium was added containing the same antipsychotic with or without 30 μM curcumin, as indicated, and the cells were incubated for additional 2 h. Finally, ^3^H-radioactivity was measured in both the cells and medium. The ^3^H-cholesterol efflux into the medium is expressed as the percentage of total radioactivity in the well (cells plus medium). Results are mean ± SEM of three independent experiments performed in duplicate. Statistical comparisons shown are curcumin versus no curcumin (* *P*<0.05, ** *P*<0.01 and *** *P*<0.001).

To determine the effects of curcumin on cholesterol efflux, HepG2 cells maintained in LPDS medium were treated with an antipsychotic (clozapine, haloperidol, risperidone, or ziprasidone) plus ^3^H-cholesterol-labeled LDL or left untreated for 16 h, washed in PBS, and incubated for 2 h with serum-free medium containing the same antipsychotic with or without curcumin. At end of the incubation, ^3^H-cholesterol was measured in both cells and media. In antipsychotic treated cells, curcumin enhanced the cholesterol efflux outside the cells (Fig 4). In separate experiments, we determined the total cholesterol in HepG2 cells at the end of incubation. In HepG2 cells incubated in the presence of LDL and an antipsychotic for 16 h, subsequent treatment with curcumin significantly reduced the total intracellular cholesterol content (Table A in S1 File). Given that cells were incubated in the absence of cholesterol acceptors (i.e. ApoA1 [apolipoprotein A1] or HDL), these results suggest that mechanism(s) different than those mediated by ABC-transporters must account for the promotion of cholesterol release by curcumin.

### Curcumin-induced secretion of endosomal/lysosomal proteins from antipsychotic-treated cells

**Fig 5:**
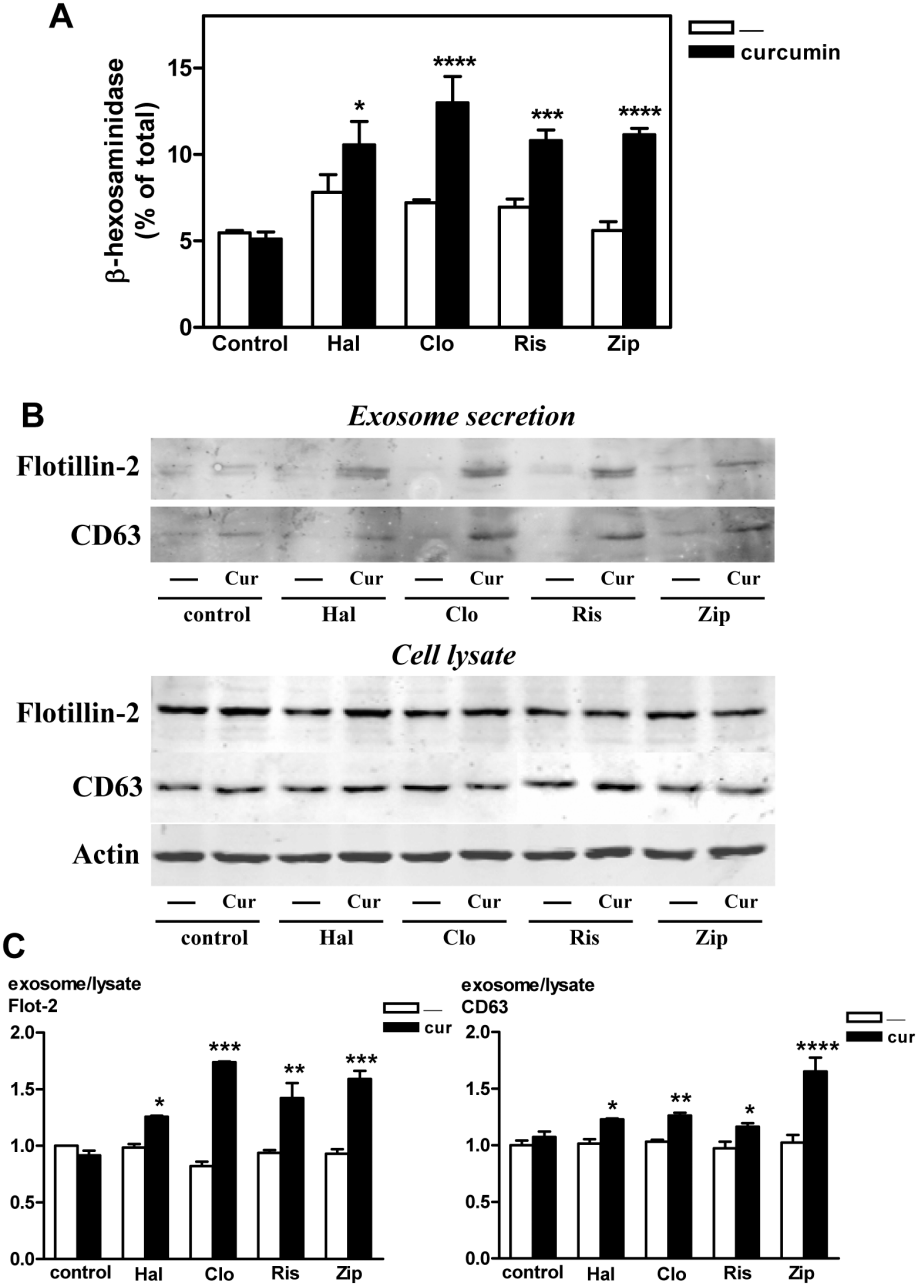
Curcumin enhances the release of lysosomal β-hexosaminidase (A) and flotillin-2- and CD63-containing exosomes (B and C) from HepG2 cells treated with antipsychotics. Cells were exposed to LDL (60 μg/ml of cholesterol) in the absence (control) or the presence of haloperidol (Hal), clozapine (Clo), risperidone (Ris), or ziprasidone (Zip) (10 μM) for 16 h. Cells were washed twice and then incubated for additional 2 h in serum-free medium containing the same antipsychotic with or without curcumin (Cur, 30 μM) as indicated. (A) At the end of the incubation, the activity of the lysosomal enzyme β-hexosaminidase was measured in both cells and medium. The enzyme activity released into the medium is expressed as the percentage of total enzyme content (cells plus medium). Results are mean ± SEM of three independent experiments performed in triplicate. (B and C) At the end of the incubation, the medium was removed, and isolated exosomes and cell lysates analyzed by Western blot. (B) Flotillin-2, CD63, and actin Western blots from exosome and HepG2 cell lysate. Results are representative of 3 independent experiments. (C) Quantitation of exosomal release of flotillin-2 (Flot-2) and CD63 from HepG2 cells. Results are mean ± SEM of three independent experiments; the control without curcumin is normalized to 1. Statistical comparisons shown are curcumin versus no curcumin (* *P*<0.05, ** *P*<0.01, *** *P*<0.001 and **** *P*<0.0001).

To determine whether curcumin induces late-endosome exocytosis in antipsychotic-treated HepG2 cells, we measured the lysosomal enzyme β-hexosaminidase in the culture media (Fig 5A). In cells treated with antipsychotic, curcumin significantly increase the release of β-hexosaminidase activity to the culture. In addition, curcumin enhanced the secretion of proteins considered as markers for exosomes, as indicated by Western blotting of lysates from HepG2 cells and isolated exosomes (Fig 5B and 5C). Treatment with curcumin significantly increased the content of both flotillin-2 and CD63 in the exosome fraction, while no significant changes in the content of these proteins were detected in cell lysates.

**Fig 6:**
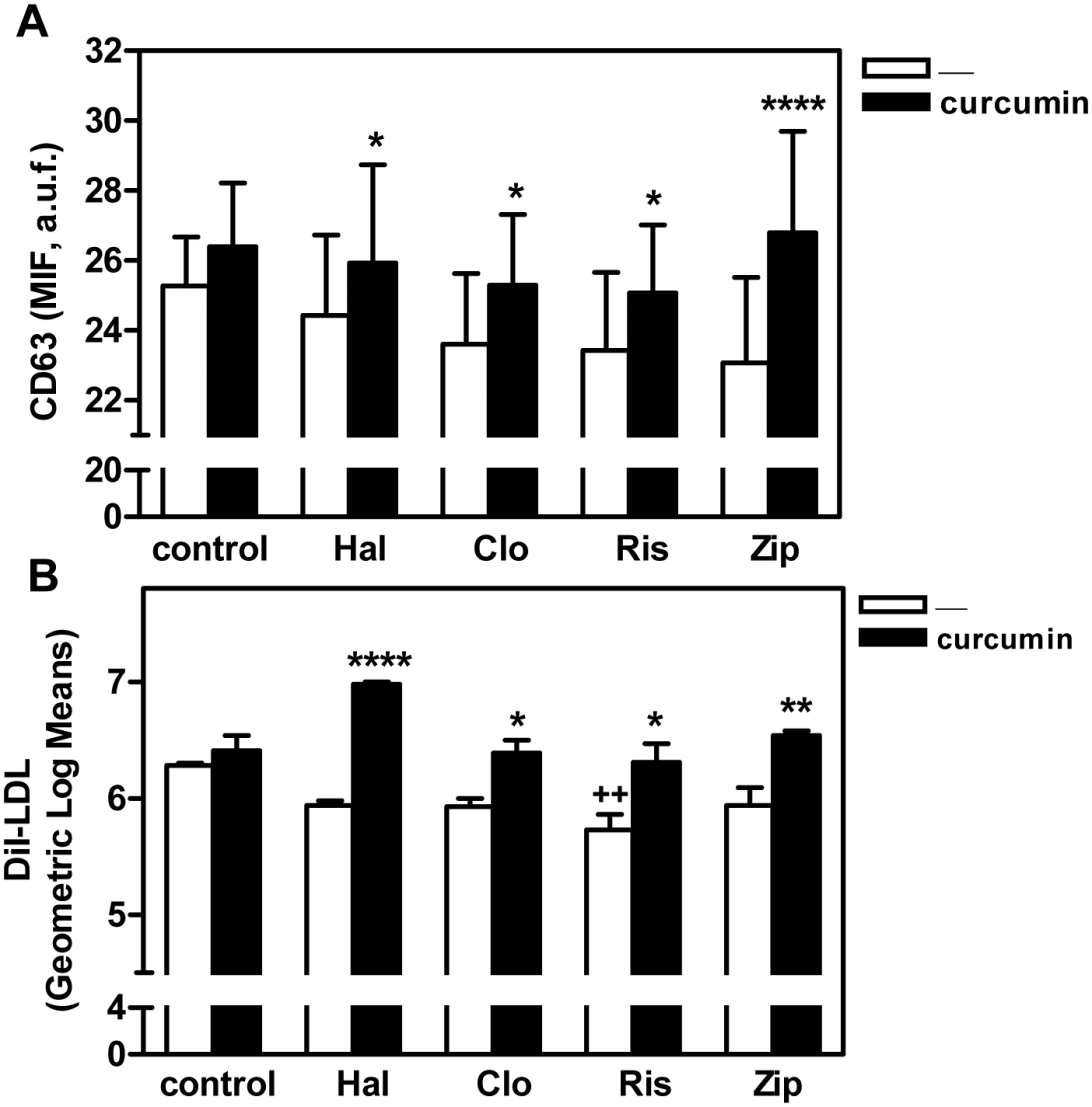
Curcumin increases CD63 surface expression and DiI-LDL content in isolated exosomes as analyzed by FACS. HepG2 cells were incubated with antipsychotics (haloperidol [Hal], clozapine [Clo], risperidone [Ris], or ziprasidone [Zip], 10 μM) and DiI-labeled LDL (60 μg/ml of cholesterol) for 16 h. Cells were washed twice and serum-free medium containing the same antipsychotics with or without 30 μM curcumin added as indicated. After a further 2-h incubation, exosomes were isolated from the medium, attached to latex beads, labeled with anti-CD63, and analyzed by FACS. The graphs represent the fluorescence of CD63 (MIF) (A) and DiI-LDL (Geometric Log means) (B) in exosomes. Results are mean ± SEM of three independent experiments. M.I.F., median intensity of fluorescence; a.u.f., arbitrary units of fluorescence. Statistical comparisons shown are curcumin versus no curcumin (* *P*<0.05, ** *P*<0.01, *** *P*<0.001 and **** *P*<0.0001).

As examined by electron microscopy, secreted exosomes had a rounded structure with a median diameter of 67.5 nm (data not shown), which is too small as to be reliably analyzed by FACS directly. Thus, for this study, isolated exosomes were fixed to aldehyde sulfate-latex beads of a size within the detection range of a flow cytometer [38,39], and exosome-bound CD63 and DiI were measured by flow cytometry. As shown in Fig 6, curcumin treatment significantly increased the release of both CD63 and DiI bound to exosomes from cells treated with antipsychotics. Moreover, curcumin also increased cholesterol levels in isolated exosomes released from antipsychotic-treated HepG2 cells compared to exosomes from control cultures as measured by GC/MS (Table 1).

**Table 1:** Cholesterol content in exosomes secreted by HepG2 cells.

	Cholesterol (ng/total extract)	
	– curcumin	+ curcumin
**Control**	119.2 ± 31.6	261.2 ± 36.2***
**Haloperidol**	145.0 ± 2.5	410.0 ± 32.5**** ^,^ ^++++^
**Clozapine**	161.3 ± 16.3	413.8 ± 6.3**** ^,^ ^++++^
**Risperidone**	97.5 ± 2.5	331.3 ± 63.8****
**Ziprasidone**	113.8 ± 8.8	546.3 ± 21.3**** ^,^ ^++++^

Cells were exposed to LDL (60 μg/ml of cholesterol) in the absence (control) or the presence of antipsychotics (haloperidol, clozapine, risperidone and ziprasidone, 10 μM) for 16 h. Cells were washed twice, and serum-free medium was added containing the same antipsychotic with or without 30 μM curcumin, as indicated, and the cells were incubated for additional 2 h. Then, exosomes were isolated from the medium and analyzed by GC/MS. Data are shown as ng of cholesterol in the total exosomes extract. Results are presented as means ± SEM of 3 independent experiments. Statistical comparisons are shown curcumin versus no curcumin

(* *P*<0.05,

** *P*<0.01,

*** *P*<0.001 and

**** *P*<0.0001) or versus control treated with curcumin (

^+^ *P*<0.05,

^++^ *P*<0.01,

^+++^ *P*<0.001 and

^++++^ *P*<0.0001).

Taken together, these results show that curcumin promotes exosome secretion from antipsychotic-treated HepG2 cells. The higher cholesterol content of these exosomes following curcumin treatment strongly indicate that curcumin promotes lipid egress from the cell, thereby alleviating endosomal/lysosomal cholesterol deposition caused by antipsychotic-induced inhibition of intracellular lipid trafficking.

### Effects of curcumin on cholesterol esterification

**Fig 7:**
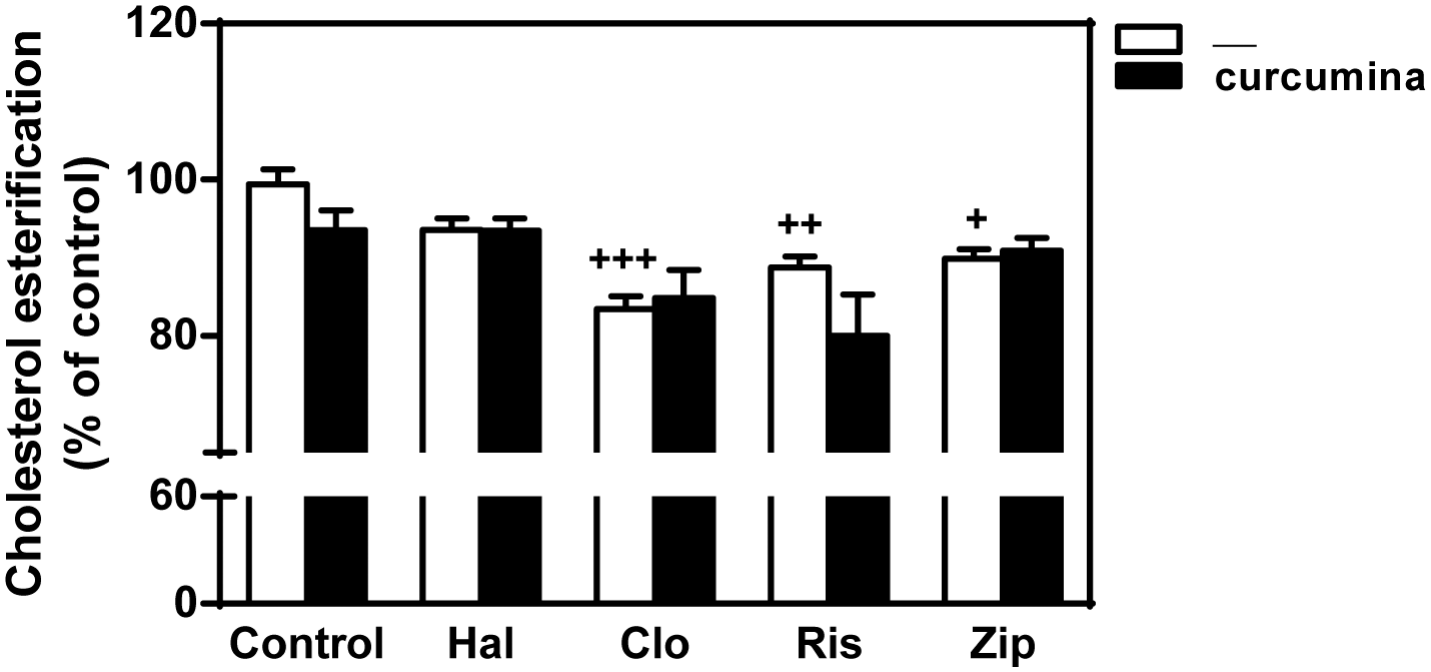
Effect of curcumin on the incorporation of [^3^H]oleic acid into cholesterylesters in HepG2 cells treated with antipsychotics. Cells were exposed to LDL (60 μg/ml of cholesterol) in the absence (control) or the presence of haloperidol (Hal), clozapine (Clo), risperidone (Ris), or ziprasidone (Zip) (10 μM) for 16 h. Cells were washed twice and then incubated for additional 2 h in serum-free medium containing the same antipsychotic with or without curcumin (30 μM) and [^3^H]oleic acid. Then, cells were collected, lipids extracted and separated by HPLC. Results are mean ± SEM of three independent experiments. Statistical comparisons shown antipsychotic versus untreated control (^+^ *P*<0.05, ^++^ *P*<0.01 and ^+++^ *P*<0.001).

Cholesterol in the ER can be esterified by acyl-coenzyme A:cholesterol acyltransferase (ACAT) for storage [14]. We determined the [^3^H]oleic acid incorporation into cholesteryl esters as a measure of cholesterol availability in the ER. As shown in Fig 7, treatment with antipsychotics reduced cholesterol esterification compared to control, an effect that was statistically significant for clozapine, risperidone and ziprasidone, but not for haloperidol. The esterification rate was not affected by curcumin, suggesting that curcumin did not increase the availability of free cholesterol in the ER. Thus, the reduction of cholesterol accumulation within the endolysosomal compartment induced by this polyphenol is achieved via exosome secretion rather than egress to the ER.

## Discussion

Prolonged treatment with antipsychotics may be accompanied by adverse effects like weight gain, dyslipidemia, and insulin resistance as shown in both patients with schizophrenia and older people with dementia [2,3]. Antipsychotics, both FGAs and SGAs, have been shown to inhibit cholesterol synthesis and to induce intracellular lipid accumulation in late endosomes in different cell lines [5,6,8]. These effects could underlie some of the metabolic adverse effects induced by prolonged treatment with antipsychotic [8]. Due to the array of deleterious consequences this may have on patients, we investigated the possibility of alleviating the intracellular LDL trafficking inhibition and lipid accumulation induced by antipsychotics. In this study, we demonstrate that the natural turmeric-derived polyphenol promotes cholesterol clearance from antipsychotic-treated cells.

We first studied the ultrastructural changes in HepG2 cells induced by antipsychotics. Both haloperidol, a FGA, and three SGAs, clozapine, risperidone, and ziprasidone, induced profound changes in subcellular organelles, including increased numbers of heterolysosomes or MVBs containing electron-dense material. This finding was in accordance with the profusion of late endolysosomes observed by immunocytochemical analysis. Strikingly, curcumin treatment reduced the number of heterolysosomes in cells treated with antipsychotics. To assess whether these phenotypic changes were due to the affectation of lipid homeostasis, we studied the intracellular fate of endocytosed DiI-labeled LDL. Treatment with antipsychotics induced the accumulation of perinuclear late endolysosomes enriched in both cholesterol and DiI, confirming previous results [8]. Addition of curcumin to the medium for just 2 hours decreased the size of granules positive for DiI, filipin, and CD63. Moreover, curcumin-treated cells exhibited abundant DiI- and filipin-positive vesicles around the cell periphery, suggesting that curcumin was promoting lipid egress by stimulating exocytosis.

The release of exosomes, which originate from intraluminal vesicles within MVBs [27], may act to remove materials accumulated in endolysosomes, including lipids and hydrolytic enzymes [31]. Strauss *et al*. (2010) reported that both oligodendroglial cells treated with U18666A and NPC1-deficient fibroblasts secrete cholesterol-containing exosomes, and proposed that exosomal release serves as a mechanism to partially alleviate intracellular cholesterol trafficking block [32]. In line with this, we found that curcumin stimulated the release of the lysosomal enzyme β-hexosaminidase into the media, as well as flotillin-2 and CD63, which both are considered as exosome markers [29,32,40]. Moreover, curcumin increased the secretion of cholesterol and LDL-derived DiI and [^3^H]-cholesterol, which was associated with the decrease of the levels of these lipids within the cells. Finally, the release of exosomes to the medium was confirmed by the identification of particles with the predicted size and morphology, as examined by electron microscopy, and the demonstration that extracellular vesicles isolated by ultracentrifugation contained CD63 on their surface, an exosome marker [40]. In cells loaded with DiI-labeled LDL, these secreted particles also contained DiI, all of which unequivocally demonstrates their endolysosomal origin.

In adipocytes and macrophages, other authors reported that curcumin stimulates cholesterol efflux, which was attributed to the upregulation of ABCA1 [24,25]. As we use a serum-free medium containing no detectable ApoA1 to assay cholesterol secretion, the contribution of this additional mechanism in our experimental conditions should be of less importance. The unequivocal demonstration that curcumin stimulates exosome secretion suggests that this mechanism promotes lipid clearance in antipsychotic-treated HepG2 cells, thus contributing to mitigate the lipid deposition phenotype. Whether other mechanisms also contribute to the lipid secretion induced by curcumin, however, can not be ruled out.

We asked whether curcumin also stimulates the egress of lysosomal cholesterol to the ER. For this we measured the cholesterol esterification by ACAT, which is dependent on the cholesterol availability in the ER [14,41]. Treatment with antipsychotics diminished the incorporation of oleic acid into cholesterol esters, which is in accordance with the inhibition of the cholesterol egress of cholesterol from the endolysosomal compartment induced by these drugs. However, curcumin did not mitigate this effect of antipsychotics, indicating that this polyphenol, at least during the studied period, did not facilitate the arrival of free cholesterol to the ER.

Previous studies by others reported the reduction of cellular cholesterol levels by effect of curcumin in cells in culture [23]. In mice fed a high-fat diet, curcumin also attenuated hepatic accumulation of cholesterol and triglycerides [42,43]. In N-methyl *N*-nitrosourea-treated mice, curcumin administration normalized total lipids, cholesterol, and phospholipids in membrane preparations from cerebrum and cerebellum [44]. Finally, curcumin also has been shown to protect against antipsychotics side effects not related to lipid metabolism, such as oro-facial dyskinesia induced by haloperidol in animal models [45,46], all showing the potential of this polyphenol for disease prevention.

The mechanism by which curcumin stimulates exosome secretion is not known.

Among other actions, curcumin has been shown to potently inhibit SERCA, thus increasing cytosolic Ca^2+^ levels [17] and also activates neutral sphingomyelinase and inhibits sphingomyelin synthase, leading to the accumulation of ceramides within the cell [47]. These two agents, intracellular calcium and ceramides, are involved in exosome release. Thus, Ca^2+^ has been shown to accumulate in the MVB upon the stimulation of the secretory process and increasing intracellular Ca^2+^ markedly enhances exosome secretion [28]. On the other side, intracellular production of ceramides has been described to stimulate exosomes generation [48]. Interestingly, the alteration of calcium homeostasis directly affects the sphingolipid trafficking inside the cell, followed by the storage of cholesterol in acidic intracellular compartment, which illustrates the connection among these factors [20]. Based on these results, it may be speculated that curcumin stimulates exosome secretion by acting on both Ca^2+^ calcium homeostasis and sphingolipid metabolism, a hypothesis that should be confirmed in future studies.

In conclusion, we show that curcumin accelerates the release of exosomes containing cholesterol from cells with impaired intracellular cholesterol trafficking due to antipsychotic treatment. The egress of cholesterol (and presumably other lipids) out of the cell ameliorated cholesterol retention within the endolysosomal compartment. This effect of curcumin may help minimize the adverse metabolic effects associated with chronic antipsychotic treatment. The low bioavailability of curcumin [49] may limit the extrapolation of present results to the in vivo condition, which deserves to be further studied and tested in clinical trials.

## Supporting Information

S1 File Fig A: Effect of different doses of curcumin on the intracellular DiI-LDL accumulation in cells treated with haloperidol. HepG2 cells were incubated with DiI-LDL (30 μg/ml of cholesterol) for 16 h in the absence (control) or the presence of haloperidol (10 μM). Then, cells were washed and serum-free medium containing the same antipsychotic without or with 5, 10, 30 or 40 μM curcumin was added and incubated for additional 2 h. Intracellular DiI-LDL was measured by flow cytometry. Results are mean ± SEM of three independent experiments performed in duplicate. M.I.F., median intensity of fluorescence; a.u.f., arbitrary units of fluorescence. Statistical comparisons shown are haloperidol versus control for each dose of curcumin (* *P*<0.05, ** *P*<0.01) and haloperidol with for each dose of curcumin versus haloperidol without curcumin (+ *P*<0.05, ++ *P*<0.01). Fig B: Electron micrographs of HepG2 cells treated with an antipsychotic (clozapine or risperidone at 10 μM) and LDL (60 μg/ml of cholesterol) for a total of 18 h. Where indicated, during the last 2 h, the cells were treated with 30 μM curcumin. Images are representative of 2 independent experiments. Lysosomes (▲), lipid inclusions (§), heterolysosomes or MVB (*). Table A: Cholesterol content in HepG2 cells. Cells were exposed to LDL (60 μg/ml of cholesterol) in the absence (control) or the presence of antipsychotics (haloperidol, clozapine, risperidone and ziprasidone, 10 μM) for 16 h. Then the medium was removed, cells were washed twice, and serum-free medium was added, supplemented or not (control) with antipsychotics, and incubation was continued for 2 h in the presence of 30 μM curcumin, as indicated. At the end of the incubation, the cells were lysed, lipids were isolated and analyzed by GC/MS. Data are shown as ng of cholesterol per mg of protein. Results are presented as means ± SEM of three independent experiments. Statistical comparisons are shown curcumin versus without curcumin (* *P*<0.05, ** *P*<0.01 and *** *P*<0.001) or versus control without curcumin (^+^ *P*<0.05).
(PDF)

## Abbreviations

ABCA1: ATP binding cassette protein A1
ACAT: acyl-coenzyme A:cholesterol acyltransferase
ApoA1: apolipoprotein A1
DiI: 1,1′-dioctadecyl-3,3,3,3′-tetramethylindocarbocyanine perchlorate
DMSO: dimethyl sulfoxide
ER: endoplasmic reticulum
FACS: fluorescence-activated cell sorting
FBS: fetal bovine serum
FGA: typical or first-generation antipsychotic
GC/MS: gas chromatography mass spectrometry
LDL: low-density lipoprotein
LDLR: LDL receptor
LPDS: lipoprotein-deficient serum
MVB: multivesicular body or heterolysosome
NPC: Niemann-Pick type C
PFA: paraformaldehyde
SERCA: sarco/endoplasmic reticulum Ca^2+^-ATPase
SGA: atypical or second generation antipsychotic
SREBP: sterol regulatory element-binding protein
