# Supplementary material for: Curcumin Mitigates the Intracellular Lipid Deposit Induced by Antipsychotics In Vitro
Source: PLoS One. 2015 Oct 30;10(10):e0141829. doi: 10.1371/journal.pone.0141829 (PMC4627744; doi:10.1371/journal.pone.0141829)
Supplement: S1 File — Fig A: Effect of different doses of curcumin on the intracellular DiI-LDL accumulation in cells treated with haloperidol. HepG2 cells were incubated with DiI-LDL (30 μg/ml of cholesterol) for 16 h in the absence (control) or the presence of haloperidol (10 μM). Then, cells were washed and serum-free medium containing the same antipsychotic without or with 5, 10, 30 or 40 μM curcumin was added and incubated for additional 2 h. Intracellular DiI-LDL was measured by flow cytometry. Results are mean ± SEM of three independent experiments performed in duplicate. M.I.F., median intensity of fluorescence; a.u.f., arbitrary units of fluorescence. Statistical comparisons shown are haloperidol versus control for each dose of curcumin (* P<0.05, ** P<0.01) and haloperidol with for each dose of curcumin versus haloperidol without curcumin (+ P<0.05, ++ P<0.01). Fig B: Electron micrographs of HepG2 cells treated with an antipsychotic (clozapine or risperidone at 10 μM) and LDL (60 μg/ml of cholesterol) for a total of 18 h. Where indicated, during the last 2 h, the cells were treated with 30 μM curcumin. Images are representative of 2 independent experiments. Lysosomes (▲), lipid inclusions (§), heterolysosomes or MVB (*). Table A: Cholesterol content in HepG2 cells. Cells were exposed to LDL (60 μg/ml of cholesterol) in the absence (control) or the presence of antipsychotics (haloperidol, clozapine, risperidone and ziprasidone, 10 μM) for 16 h. Then the medium was removed, cells were washed twice, and serum-free medium was added, supplemented or not (control) with antipsychotics, and incubation was continued for 2 h in the presence of 30 μM curcumin, as indicated. At the end of the incubation, the cells were lysed, lipids were isolated and analyzed by GC/MS. Data are shown as ng of cholesterol per mg of protein. Results are presented as means ± SEM of three independent experiments. Statistical comparisons are shown curcumin versus without curcumin (* P<0.05, ** P<0.01 and *** P< [file pone.0141829.s001.pdf]

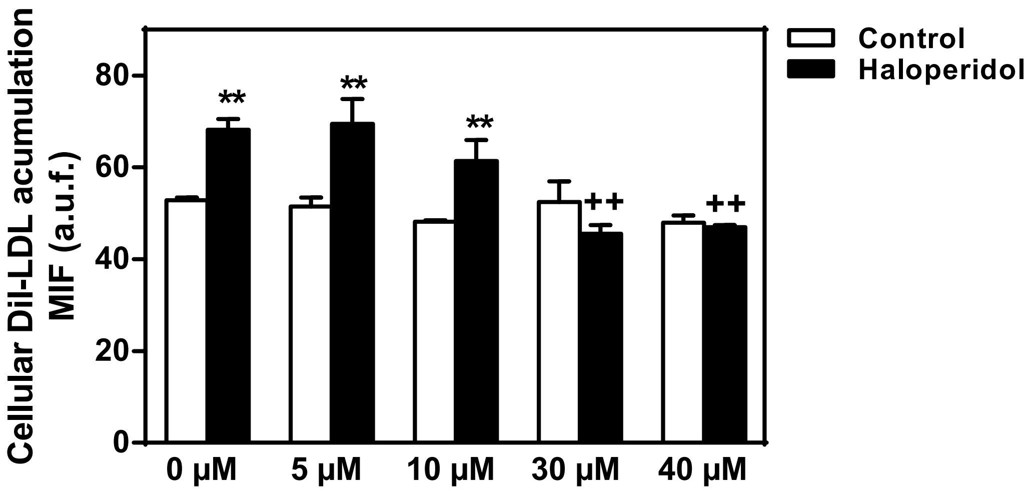

Figure A. Effect of different doses of curcumin on the intracellular DiI-LDL accumulation in cells treated with haloperidol. HepG2 cells were incubated with DiI-LDL (30 µg/ml of cholesterol) for 16 h in the absence (control) or the presence of haloperidol (10 µM). Then, cells were washed and serum-free medium containing the same antipsychotic without or with 5, 10, 30 or 40 µM curcumin was added and incubated for additional 2 h. Intracellular DiI-LDL was measured by flow cytometry. Results are mean ± SEM of three independent experiments performed in duplicate. M.I.F., median intensity of fluorescence; a.u.f., arbitrary units of fluorescence. Statistical comparisons shown are haloperidol versus control for each dose of curcumin (* $P<0.05$, ** $P<0.01$) and haloperidol with for each dose of curcumin versus haloperidol without curcumin (+ $P<0.05$, ++ $P<0.01$).

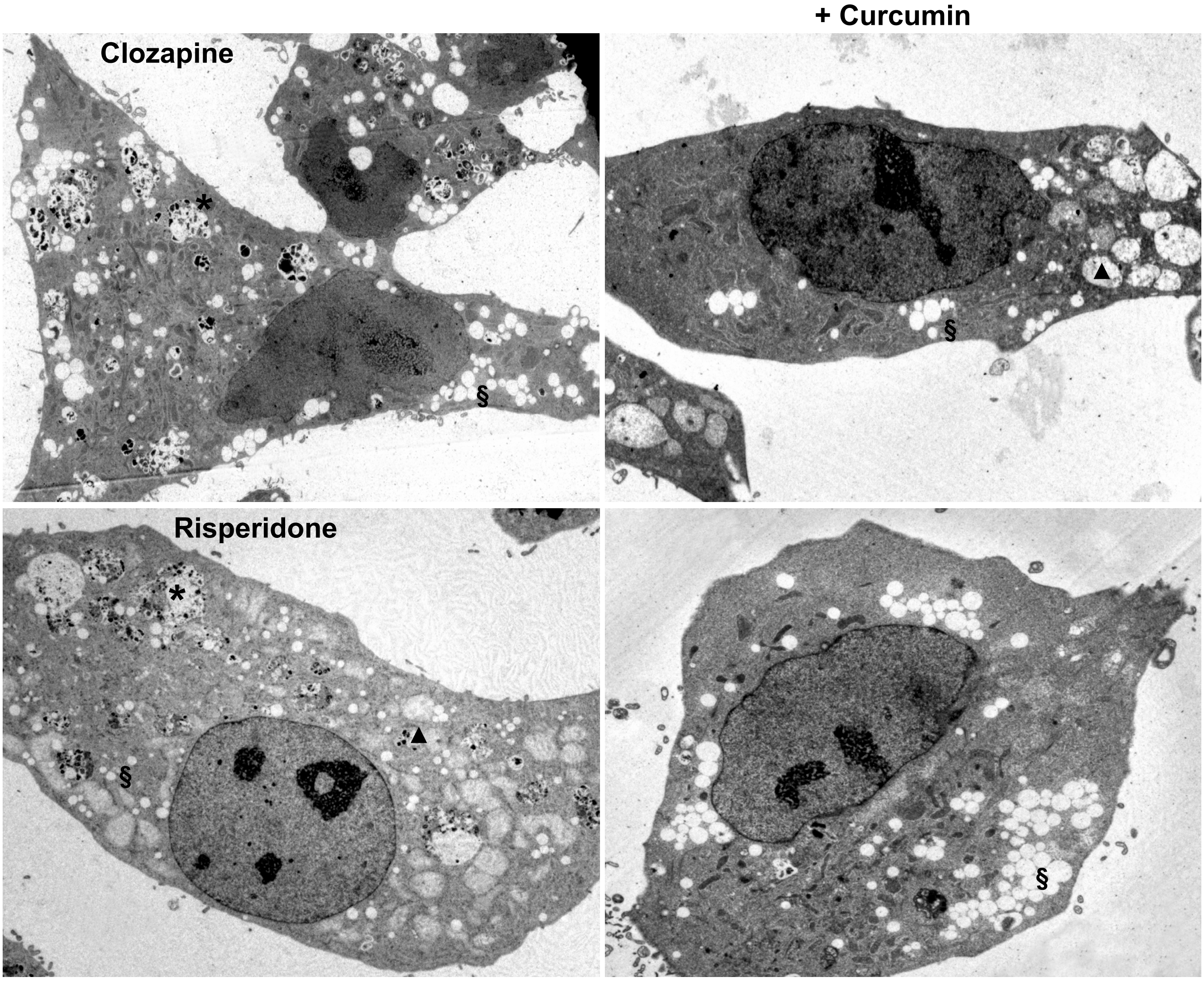

Figure B. Electron micrographs of HepG2 cells treated with an antipsychotic (clozapine or risperidone at 10 µM) and LDL (60 µg/ml of cholesterol) for a total of 18 h. Where indicated, during the last 2 h, the cells were treated with 30 µM curcumin. Images are representative from 2 independent experiments. Lysosomes (▲), lipid inclusions (§), heterolysosomes or MVB (*).

Table A. Cholesterol content in HepG2 cells.

	Cholesterol (ng/mg protein)	
	**– curcumin**	**+ curcumin**
**Control**	21944 ± 601	21597 ± 2266
**Haloperidol**	25844 ± 2374^{+}	18745 ± 636***
**Clozapine**	24635 ± 630	18411 ± 1071**
**Risperidone**	25368 ± 1423	21378 ± 980*
**Ziprasidone**	25922 ± 585^{+}	20023 ± 1396**

Cells were exposed to LDL (60 µg/ml of cholesterol) in the absence (control) or the presence of antipsychotics (haloperidol, clozapine, risperidone and ziprasidone, 10 µM) for 16 h. Then the medium was removed, cells were washed twice, and serum-free medium was added, supplemented or not (control) with antipsychotics, and incubation was continued for 2 h in the presence of 30 µM curcumin, as indicated. At the end of the incubation, the cells were lysated, lipids were isolated and analyzed by GC/MS. Data are shown as ng of cholesterol per mg of protein. Results are presented as means ± SEM of three independent experiments. Statistical comparisons are shown curcumin versus without curcumin (* $P<0.05$, ** $P<0.01$ and *** $P<0.001$) or versus control without curcumin ($^{+}$ $P<0.05$).
